# Synthesis of ellagic acid glucoside using glucansucrase from *Leuconostoc* and characterization of this glucoside as a functional neuroprotective agent

**DOI:** 10.1186/s13568-021-01265-x

**Published:** 2021-07-21

**Authors:** Hyejin Yu, Hana Jeong, Kwang-Yeol Yang, Jeong-Yong Cho, In Ki Hong, Seung-Hee Nam

**Affiliations:** 1 Institute of Agricultural Science and Technology, Chonnam National University, Gwangju, 61186 South Korea; 2 Department of Applied Biology Graduate School, Chonnam National University, Gwangju, 61186 South Korea; 3 Division of Food Technology & Biotechnology, Chonnam National University, Gwangju, 61186 South Korea; 4 Kolmar Korea R&D Center, 61, Heolleung-ro 8-gil, Seocho-gu, Seoul, 06800 Republic of Korea

**Keywords:** Ellagic acid, Glucansucrase, *Leuconostoc mesenteroides*, Transglucosylation, Brain protective effect

## Abstract

Ellagic acid glucoside was synthesized via transglucosylation using sucrose and glucansucrase derived from *Leuconostoc mesenteroides* B-512 FMCM. After such enzymatic synthesis, the product was purified by 50% ethyl acetate fraction and C18 column chromatography. Modification of ellagic acid glucoside was verified by LC–MS/MS at m/z 485.1 (M + Na)^−^ and m/z 531.1 (M + 3Na)^−^. The yield of ellagic acid glucoside was 69% (3.47 mM) by response surface methodology using 150 mM sucrose, 300 mU/mL glucansucrase, and 5 mM ellagic acid. The synthesized ellagic acid glucoside showed improved water solubility, up to 58% higher brain nerve cell (SH-SY5Y) protective effect, threefold higher cortisol reducing effect, and fourfold stronger inhibitory effect on acetylcholinesterase (AChE) than ellagic acid. These results indicate that ellagic acid glucoside could be used as a neuroprotective agent.

**Supplementary Information:**

The online version contains supplementary material available at 10.1186/s13568-021-01265-x.

## Introduction

Ellagic acid is a phenolic compound and also, dilactone dimer form of gallic acid. It is contained in many fruits such as strawberries, blackberries, raspberries, and pomegranates (Yoshimura et al. 2005). Previous studies have shown that ellagic acid can protect brain cells against stress, improve cognitive ability, and prevent Alzheimer's disease (Farbood et al. 2015). Anti-mutagenic, anti-inflammatory, anti-cancer, and antioxidant effects of ellagic acid have also been reported (Priyadarsini et al. 2002). In addition, ingesting pomegranate extract containing 100 mg of ellagic acid per day for 4 weeks could improve skin pigmentation caused by UV rays for human (Kasai et al. 2006).

Because of these effects, pomegranate extract juice is sold in Korea by promoting that it contains a lot of ellagic acid. However, pharmaceutical and cosmetic applications of ellagic acid are limited due to its extremely low water solubility.

To solve these problems, enzymatic modification of phenolic compounds using transglycosylation enzymes such as glucansucrase has attracted attention because such modifications could improve their physical, chemical, and physiological properties (Moon et al. 2007b). Glucansucrase not only can catalyze the synthesis of dextran from sucrose by transglucosylation, but also can catalyze the transfer of a glucose unit to other carbohydrates or phenolic compounds via glycosidic linkages (Moon et al. 2006; Robyt et al. 2008). Enzymatic transglycosylations by dextransucrase from *Leuconostoc mesenteroides* have been used to improve bioactivities and functional properties of various compounds (Seo et al. 2009). According to our previous studies, transglycosylated gallic acid shows higher anti-lipid peroxidation and stronger inhibition activities against tyrosinse than gallic acid (Nam et al. 2017b). Caffeic acid glucoside shows stronger inhibition on colon cancer cell growth than caffeic acid without transglycosylation (Nam et al. 2017a).

In this study, ellagic acid glucoside was synthesized from sucrose, ellagic acid, and glucansucrase from *L. mesenteroides* B-512 FMCM. The produced ellagic acid glucoside was purified by column chromatography and confirmed by LC–MS/MS. Optimal production conditions for ellagic acid glucoside were determined by response surface methodology. Moreover, functional properties of ellagic acid glucoside were investigated to determine its potential functions as a health supplement, including its antioxidant, brain cell protective, anti-stress, and acetylcholinesterase (AChE) inhibition activities.

## Materials and methods

### Materials

Ellagic acid, silica gel, sucrose, glucose, maltose, 3-(4,5-dimethylthiazol-2-yl)-2,5-diphenyl-tetrazolium bromide (MTT), and 1,1-diphenyl-2-picrylhydrazyl (DPPH) were purchased from Sigma-Aldrich (St. Louis, MO, USA). All chemical reagents were of analytical grade and purchased from commercial sources.

### Enzyme preparation

Glucansucrase (EC 3.2.1.11) for transglycosylation was obtained from *L. mesenteroides* B-512 FMCM (KCCM 11728P), grown on LM medium with 2% (w/v) glucose as previously described (Moon et al. 2007a). Fermented culture was collected, centrifuged, and concentrated using 30 K hollow fibers (Millipore, Bedford, MA, USA). Its enzyme activity was measured at 28 °C using 100 mM sucrose as a substrate in 20 mM sodium–acetate buffer (pH 5.2) for various reaction time. Reactant aliquots were spotted onto a thin layer chromatography (TLC) silica gel 60 plate (Merck, Darmstadt, Germany) and developed in acetonitrile/water (85:15, v/v) solution. The TLC plate could be visualized by dipping into 0.03% (w/v) *N*-(1-naphthyl)-ethylenediamine and 5% (v/v) H_2_SO_4_ in methanol followed by heating at 120 °C for 10 min. The amount of released fructose was measured using an NIH densitometry Image Program (http://rsb.info.nih.gov/nih-image) with a standard compound. One unit (U) was defined as the amount of enzyme that caused the release of 1 μmol of fructose per minute at 28 °C in 20 mM sodium acetate buffer (pH 5.2).

### Synthesis, extraction, purification, and identification of ellagic acid glucoside

The reaction mixture (250 mL), which consisted of 10 mM ellagic acid, 355 mM sucrose, and B-512 FMCM glucansucrase (0.65 units/mL), was incubated in 20 mM sodium acetate (pH 5.2) at 28 °C for 6 h and boiled for 5 min to inactivate the enzyme reaction. Glucosylated ellagic acid was confirmed by TLC plate analysis (Merck, Darmstadt, Germany) at room temperature. The reaction mixture was spotted onto a TLC plate and developed with ethyl acetate/acetic acid/water (3:1:1, v/v/v) using ellagic acid, fructose, and sucrose as standard materials. The developed plate was observed at UV 254 nm and visualized with 0.03% (w/v) *N*-(1-naphthyl)-ethylenediamine-H_2_SO_4_ solution as described previously in “Enzyme preparation” section*.*

The reaction mixture was separated with 50% ethyl acetate to obtain glucosylated ellagic acid from upper layer. The upper layer solution was further concentrated under vacuum using a rotary evaporator (EYELA, Tokyo, Japan) at 47 °C and re-dissolved in 50% methanol (v/v, 50 mL). The sample was loaded on the top of a C18 silica gel column (5.0 × 50 cm). After removing remaining sugars with distilled water (total of 500 mL; flow rate at 1 mL/min), ellagic acid glucoside was gradually eluted with 10–100% (v/v) methanol. The eluted sample solution was further purified by HPLC on a PDA-MD2015 instrument (JASCO, Kyoto, Japan) using the following conditions: μ-Bondapak C18-reverse-phase column (10 μm, 300 × 19 mm, Waters, Milford, MA, USA); 0.1% formic acid in distilled water (mobile phase A) and 0.1% formic acid in methanol (mobile phase B); flow rate at 0.9 mL/min; oven temperature at 40 °C; and detection wavelength at 254 nm. Molecular mass and chemical structure of the product were determined with an LC–MS/MS Synapt HDMS system (Waters, Milford, MA, USA) through electrospray ionization tandem mass spectrometry.

### Optimization of ellagic acid glucoside production

The condition of ellagic acid glucoside synthesis was optimized using response surface methodology (RSM). Experimental data were applied via the response surface regression procedure with the second-order polynomial equation as follows (Khuri and Mukhopadhyay 2010): *Y* = β_0_ + β_1_x_1_ + β_2_x_2_ + β_3_x_3_ + β_11_x_1_^2^ + β_22_x_2_^2^ + β_33_x_3_^2^ + β_12_x_1_x_2_ + β_13_x_1_x_3_ + β_23_x_2_x_3_. Statistical analyses of the experimental design were performed using Design Expert 6.0.11 software (SAS Institute Inc., Cary, NC, USA). Fit quality for the model equation was indicated by coefficient of determination (R^2^) or adjusted R^2^ representing the fitness of the polynomial model equation. Preliminary experiments were performed to optimize conditions for ellagic acid glucoside production: glucansucrase from *L. mesenteroides*, 61–1, 239 mU; sucrose, 10–700 mM; and ellagic acid, 0.1–25.1 mM.

### Water solubility and antioxidant activity

In an Eppendorf tube, ellagic acid and ellagic acid glucoside were weighed at concentrations of 1, 5, and 10 mM. The precipitate was observed by adding water and sonicating for 1 h. Antioxidant activities of ellagic acid and ellagic acid glucoside were detected using 1-diphenyl-2-picrylhydrazyl (DPPH). Samples containing 0.01–1.0 mM solutions of ellagic acid and ellagic acid glucoside in ethanol were allowed to react with 100 mM DPPH solution for 10 min at 25 °C. Absorbance values were then obtained at 517 nm using a microplate reader (Molecular Devices, Sunnyvale, CA, USA). Radical scavenging activity was expressed as the percentage of inhibited DPPH radical concentration against ascorbic acid as a reference compound. The value of IC_50_ was designated as the concentration of a compound that resulted in 50% reduction in DPPH radicals.

### Brain cell protective and anti-stress effects

SH-SY5Y human neuroblastoma cells (KCLB 22266, Korean Cell Line Bank, Seoul, Korea) were used to determine the neuronal protective effect. These cells were cultured in RPMI-1640 medium supplemented with 10% fetal bovine serum (Sigma-Aldrich, St. Louis, MO, USA) and 1% antibiotic–antimycotic (Sigma-Aldrich, St. Louis, MO, USA) in a humidified atmosphere with 5% CO_2_ at 37 °C. These cells were then plated onto 96-well plates at a density of 10^4^–10^6^ cell/mL of medium. After 24 h, they were treated with different concentrations (1–100 μM) of ellagic acid and its glucoside. After treatment with 100 mM glutamate, cell viability was measured by MTT assay. As a positive control, 1 and 10 μM theanine was used. The absorbance was measured at 570 nm wavelength to investigate the effect of stress protection based on cell viability.

In order to quantify the anti-stress effect, the supernatant of medium solution was used after centrifugation. The experiment was performed at 4 °C. The anti-stress effect was measured using a Cortisol ELISA Kit (Calbiotech Inc., Los Angeles, CA, USA). Absorbance at 450 nm was measured using a microplate coated with cortisol MAb (monoclonal antibody).

### Acetylcholinesterase (AChE) inhibition activity

SH-SY5Y cells were treated with 100 mM glutamate to induce stress as previously described. After treatment with ellagic acid and its glucoside at two concentrations (50 and 100 μM), the supernatant of the medium was collected after centrifugation. Anti-dementia effects of ellagic acid and its glucoside were measured based on their inhibition activity on AChE enzyme known to degrade acetylcholine, a neurotransmitter, using Acetylcholinesterase (AChE) Inhibition Assay Kit (Sigma-Aldrich, St. Louis, MO, USA).

### Statistical analysis

The data were statistically processed using one-way analysis of variance (ANOVA). The experiments were repeated in triplicate and expressed as mean and standard deviation. Comparison of the difference in values between different groups was done through Duncan’s multiple-range test using SPSS program (SPSS version 23.0 for windows, SPSS Inc., Chicago, IL, USA). The data on functional evaluation were assessed using the Student’s *t*-test. Values of *p* < 0.05 were considered significant.

## Results

### Synthesis, purification, and identification

**Fig. 1:**
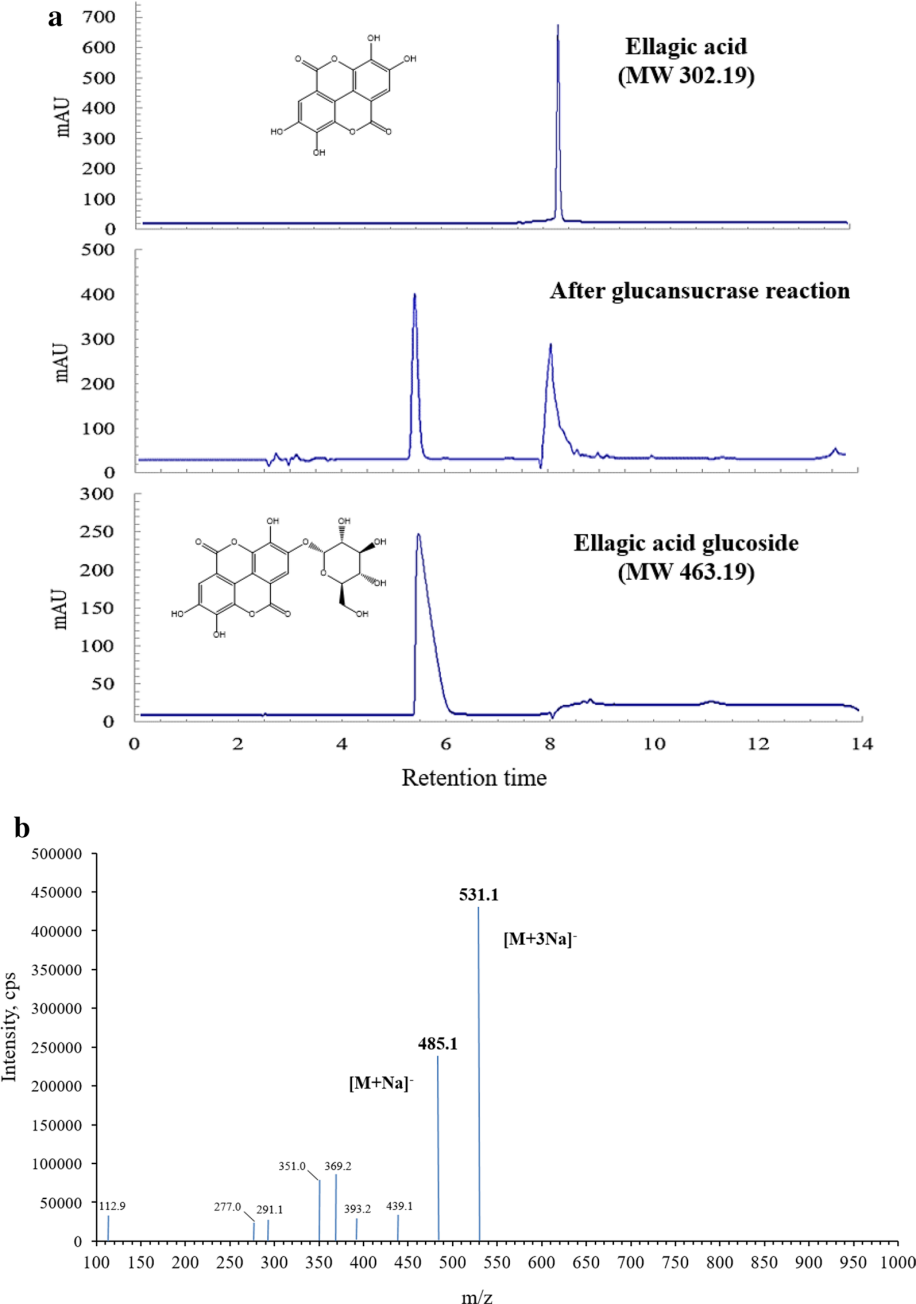

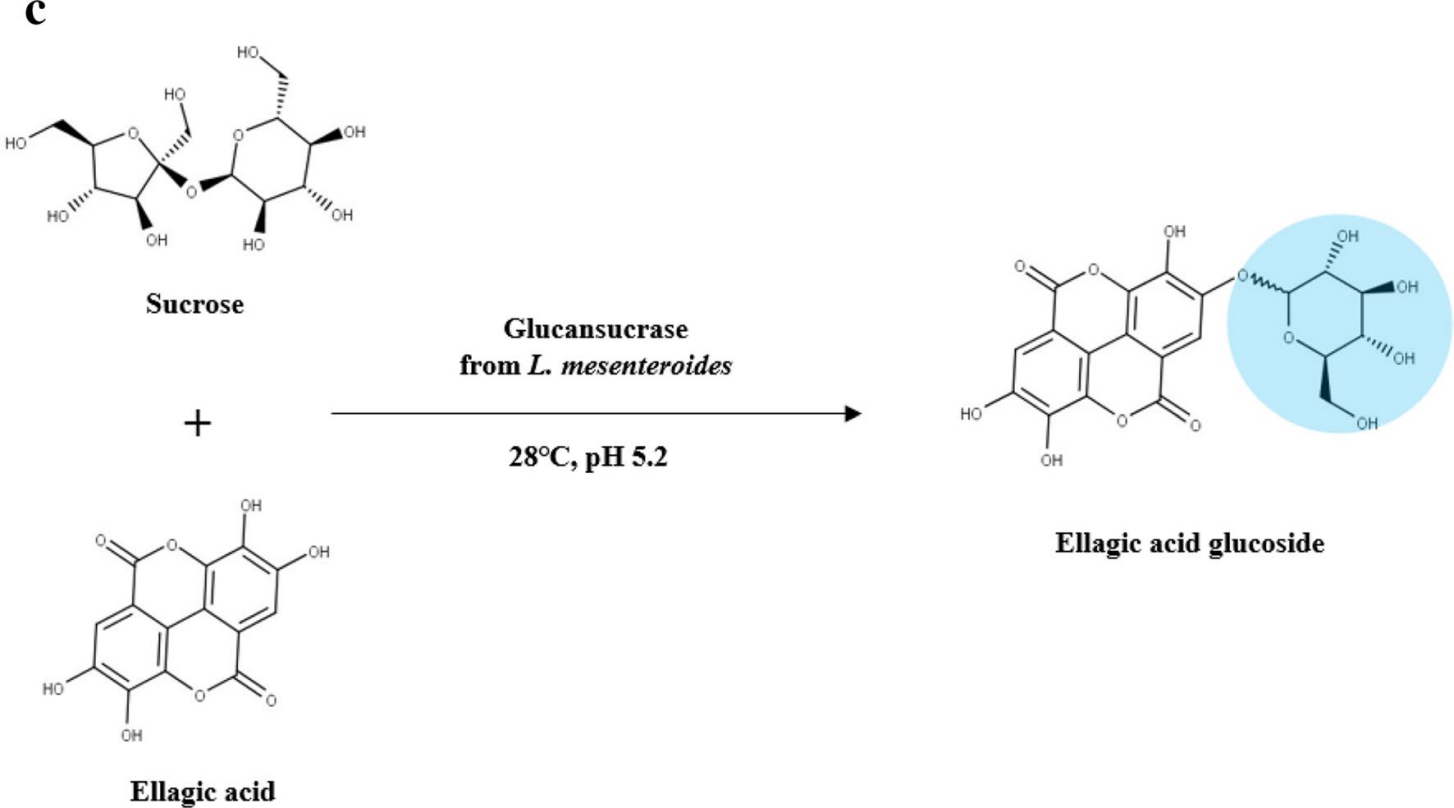
HPLC chromatogram, mass spectrum, and schematic diagram of the reaction of ellagic acid and ellagic acid glucoside after column chromatography purification. Ellagic acid standard, after the reaction with glucansucrase, and purified ellagic acid glucoside (**a**), LC–MS/MS spectrum of ellagic acid glucoside (**b**), and a schematic diagram of the reaction (**c**)

The activity of glucansucrase from *L. mesenteroids* B-12FMCM was 10.5 U/mg at 28 °C under condition of 100 mM sucrose as substrate in 20 mM sodium–acetate buffer (pH 5.2). Ellagic acid glucoside was obtained from a solution containing 10 mM ellagic acid, 355 mM sucrose, and 0.65 U/mL glucansucrase. Ellagic acid glucoside was detected as a reaction product of glucansucrase with ellagic acid and sucrose by HPLC (Fig. 1a). The reaction mixture was separated by ethyl acetate partitioning, which removed unreacted hydrolyzed carbohydrates or enzymes present in the lower layer, whereas ellagic acid and ellagic acid glucoside were concentrated in the upper layer. Furthermore, C18 column chromatography was used to purify the products. The column was washed with water to remove remained sugars or enzyme followed by elution with 10–100 (v/v) methanol gradient to obtain products with 50–60% methanol. The number of glucose units attached to the synthesized products was verified by LC–MS/MS. Molecular ions of ellagic acid glucoside were observed at m/z 485.1 (M + Na)^−^ and 531.1 (M + 3Na)^−^ (Fig. 1b). When a glucose moiety was added to ellagic acid, the molecular weight of the compound was increased to the expected structure via a glycosidic linkage (Fig. 1c). This study revealed that glucansucrase from *L. mesenteroides* B-512 FMCM could be used to synthesize glycoside linkage for a phenolic compound like other previous experiments (Nam et al. 2017a, b).

### Optimum ellagic acid glucoside synthesis

**Fig. 2:**
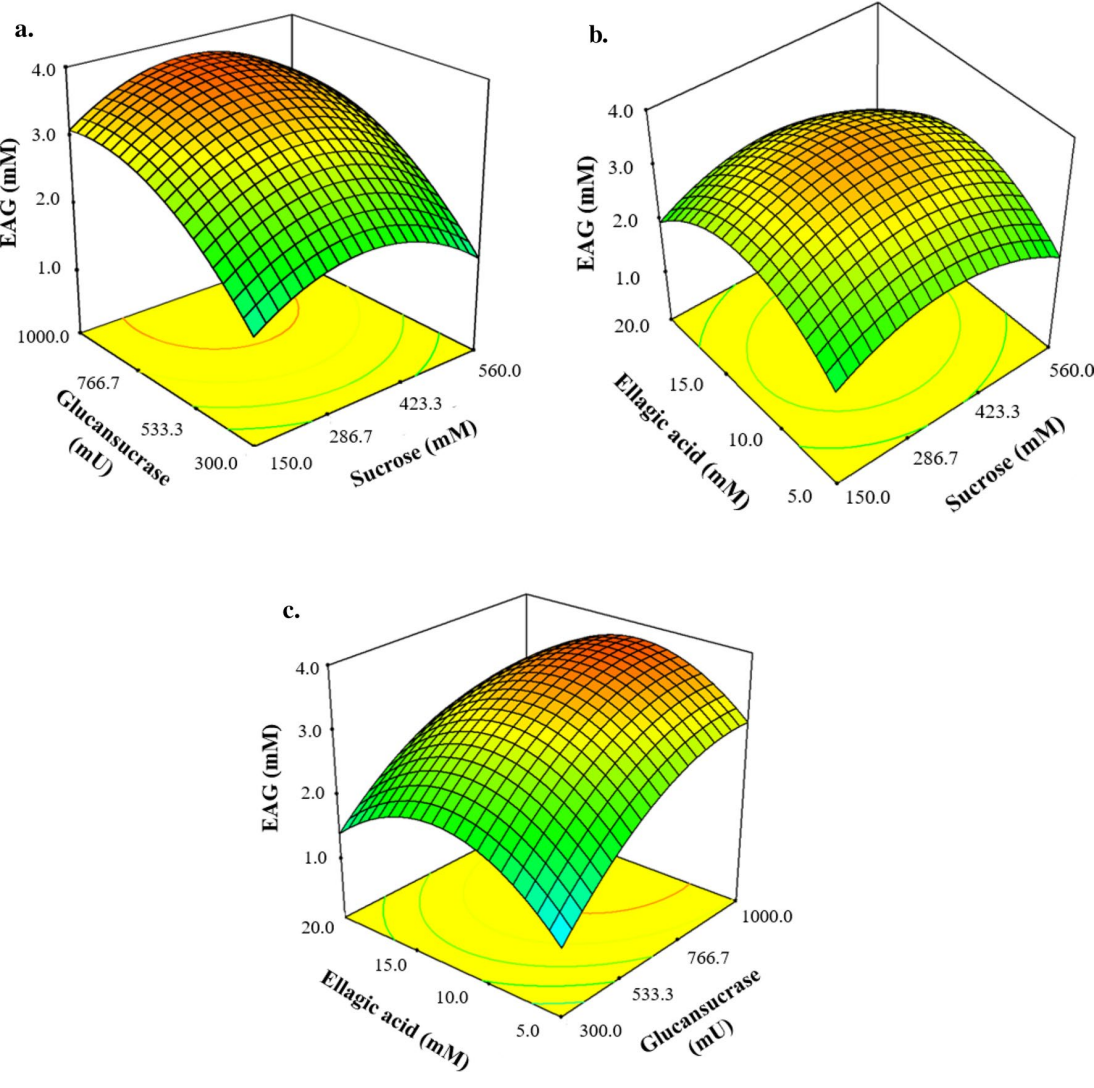
Response surface and contour plots of ellagic acid glucoside (EAG) production. Mutual interactions between glucansucrase and sucrose (**a**), between ellagic acid and sucrose (**b**), and between ellagic acid and glucansucrase (**c**) are shown. The synthesis of ellagic acid glucoside was optimized using different concentrations of glucansucrase from *L. mesenteroides* (61–1239 mU/mL), sucrose (10–700), and ellagic acid (0.1–25.1 mM)

The effect of three variables (sucrose concentration, glucansucrase unit, and ellagic acid concentration) on the yield of ellagic acid glucoside was determined. A total of 20 experiments were performed to investigate the interaction of these variables to ellagic acid glucoside synthesis. The synthesis of ellagic acid glucoside was optimized by CCD matrix using actual and predicted values as shown in Table 1. Interactions of these variables were evaluated by RSM within the range from − 1.682 to + 1.682 (Additional file 1: Table S1). Ellagic acid synthesis using glucansucrase was expressed with the following regression equation:

$$\begin{aligned} {\text{Y}} & = - 4.630 + 0.011{\text{X}}_{1} + 0.008{\text{X}}_{2} + 0.443{\text{X}}_{3} + 0.0000004{{\text{X}}_{1}}^{2} - 0.000008{{\text{X}}_{2}}^{2} \\ & \quad - 0.00004{{\text{X}}_{3}}^{2} - 0.00001{\text{X}}_{1} {\text{X}}_{2} - 0.000004{\text{X}}_{1} {\text{X}}_{3} - 0.016{\text{X}}_{2} {\text{X}}_{3} \\ \end{aligned}$$

where X_1_ was the sucrose concentration (mM), X_2_ was the glucansucrase unit (mU/mL), and X_3_ was the ellagic acid concentration (mM). R^2^ value from this regression equation was 0.82, which explained 82% of the variation in the response (Additional file 1: Table S2). If the R^2^ value is greater than 0.8, a close correlation is considered. An adequate precision value is indicative of a signal-to-noise ratio index. If the value is higher than 4, it indicates proper prerequisites for a good fitting model. That value of this model was 5.56, suggesting that navigation of the design space of the model was capable. The predicted value of ellagic acid glucose production was 3.47 mM and the experimental value was 3.51 ± 0.38 mM with 355 mM sucrose, 650 mU/mL glucansucrase, and 12.5 mM ellagic acid, showing similarities between the predicted value and the observed value of ellagic acid glucoside production. The optimum yield for ellagic acid glucoside was 3.47 mM or 69% by reacting 300 mU/mL glucansucrase with 5 mM ellagic acid and 150 mM sucrose (Fig. 2).

**Table 1:** Central composite design matrix for the experiment and predicted responses for the synthesis of ellagic acid glucoside

Run no.	Coded levels			Ellagic acid glucoside synthesis (mM)	
	X_1_	X_2_	X_3_	Actual	Predicted
1	150	300	5.0	0.97	0.47
2	560	300	5.0	0.75	0.36
3	150	1000	5.0	2.88	2.27
4	560	1000	5.0	2.70	2.30
5	150	300	20.0	1.45	0.82
6	560	300	20.0	1.08	0.67
7	150	1000	20.0	2.76	2.12
8	560	1000	20.0	2.62	2.10
9	10.2	650	12.5	0.72	1.65
10	699.8	650	12.5	1.02	1.55
11	355	61.4	12.5	0.01	0.66
12	355	1238.6	12.5	2.58	3.38
13	355	650	0.1	0.21	0.83
14	355	650	25.1	0.13	0.96
15	355	650	12.5	4.09	3.47
16	355	650	12.5	3.10	3.47
17	355	650	12.5	3.49	3.47
18	355	650	12.5	3.27	3.47
19	355	650	12.5	3.83	3.47
20	355	650	12.5	3.27	3.47

Y =  − 4.630 + 0.011X_1_ + 0.008X_2_ + 0.443X_3_ + 0.0000004X_1_^2^ − 0.000008X_2_^2^ − 0.00004X_3_^2^ − 0.00001 X_1_X_2_ − 0.000004X_1_X_3_ − 0.016X_2_X_3_

### Enhancement of solubility in water

**Fig. 3:**
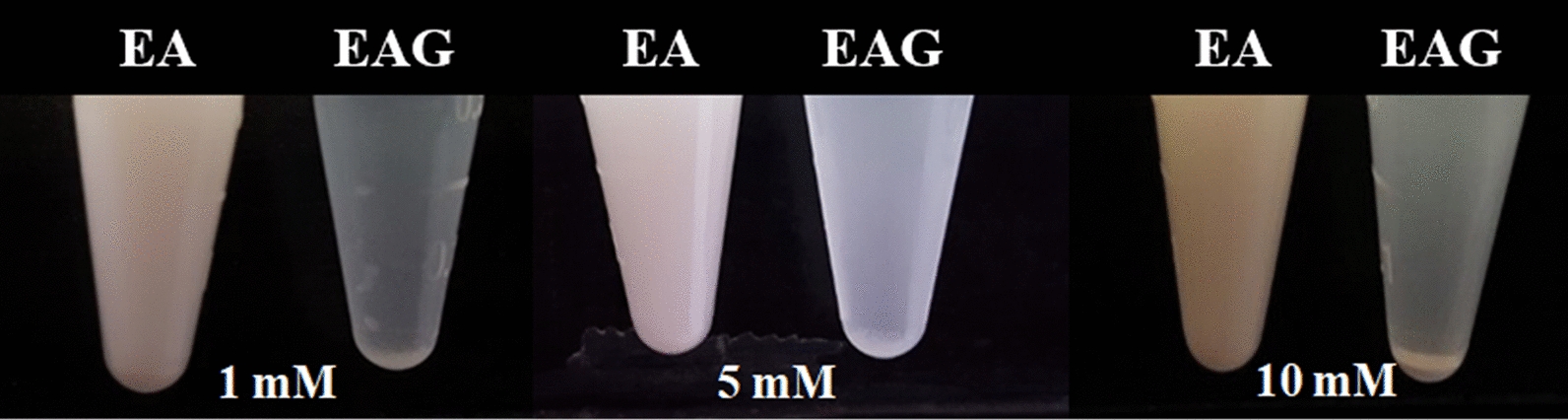
Water solubility comparison of ellagic acid (EA) and ellagic acid glucoside (EAG) at concentrations of 1, 5, and 10 mM

Ellagic acid is hardly soluble in water, although it could be dissolved up to 5 mM after transglycosylation (Fig. 3). Such enhancement of its water solubility indicates that attached glucosyl residue could positively affect its water solubility. These results are consistent with those of our previous studies demonstrating a higher solubility of caffeic acid or quercetin after glycosylation as compared to a nonglycosylated state (Nam et al. 2017a; Moon et al 2007a).

### Antioxidant activity of ellagic acid

**Fig. 4:**
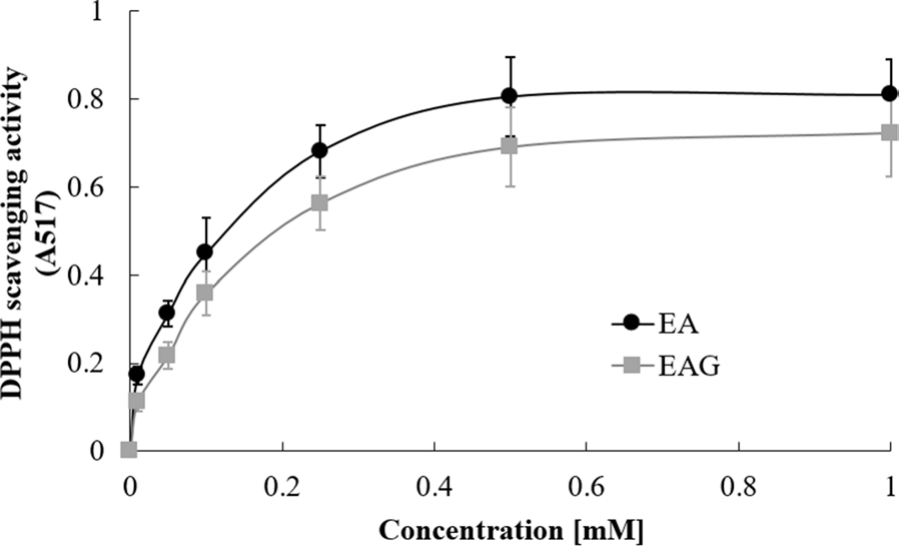
DPPH radical-scavenging activities of EA and EAG at concentrations of 0, 0.01.0.05, 0.1, 0.25, 0.5, and 1.0 mM. The reaction was monitored at 517 nm. Data are reported as mean ± SD of three separate experiments

Antioxidant activities of ellagic acid and its glucoside were determined by DPPH scavenging assay. Results are shown in Fig. 4. IC_50_ value of ellagic acid glucoside was 0.27 mM, which was higher than that of ellagic acid (IC_50_ = 0.18 mM). Because a lower IC_50_ value means a better antioxidant, transglycosylation of ellagic acid could not improve its antioxidant activity. These results indicate that the binding of glucose or sugar moiety can reduce the antioxidant capacity of compounds, consistent with our previous studies (Nam et al. 2017a, b).

### Brain cell protective, anti-stress, and anti-dementia effects of ellagic acid

**Fig. 5:**
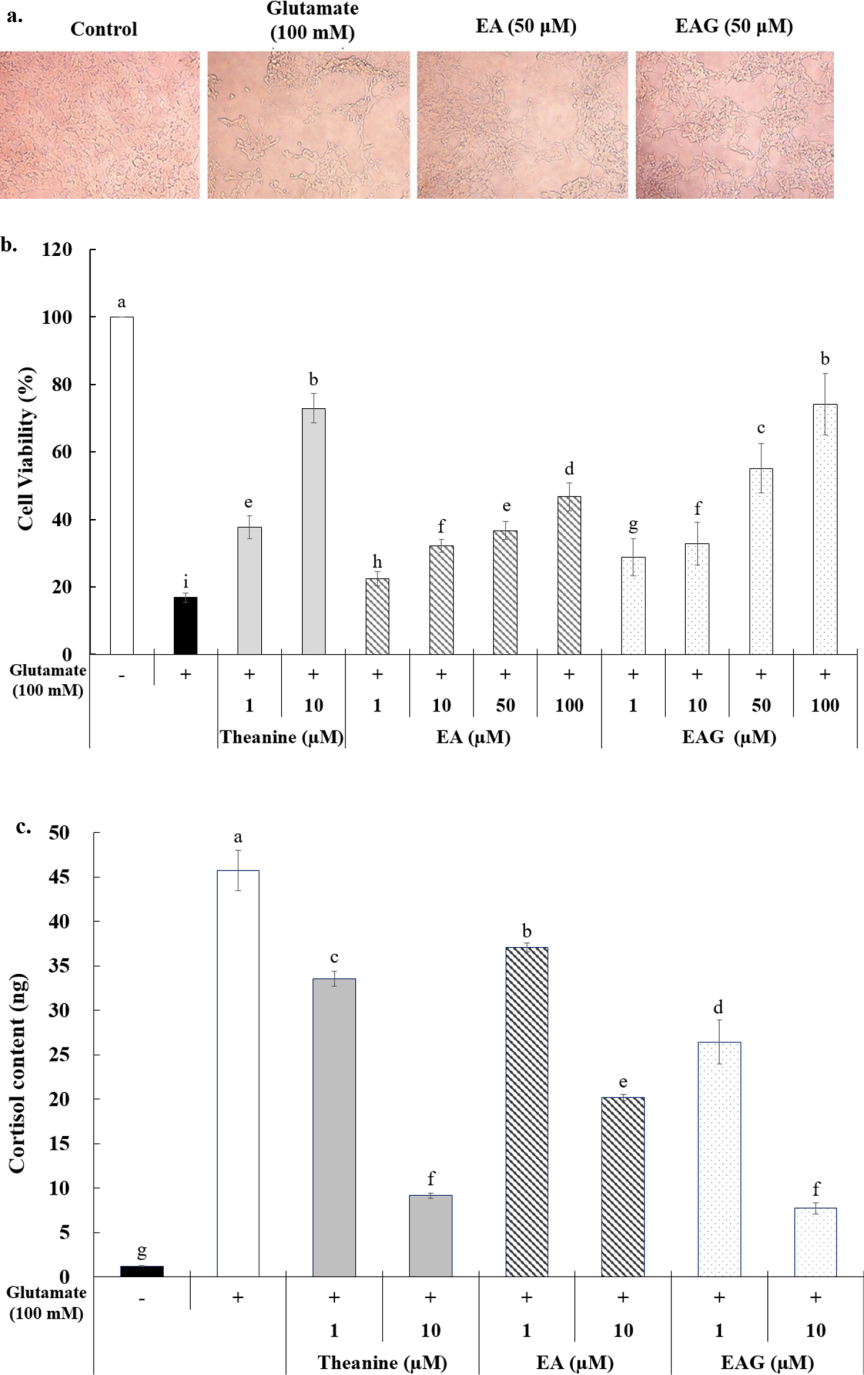

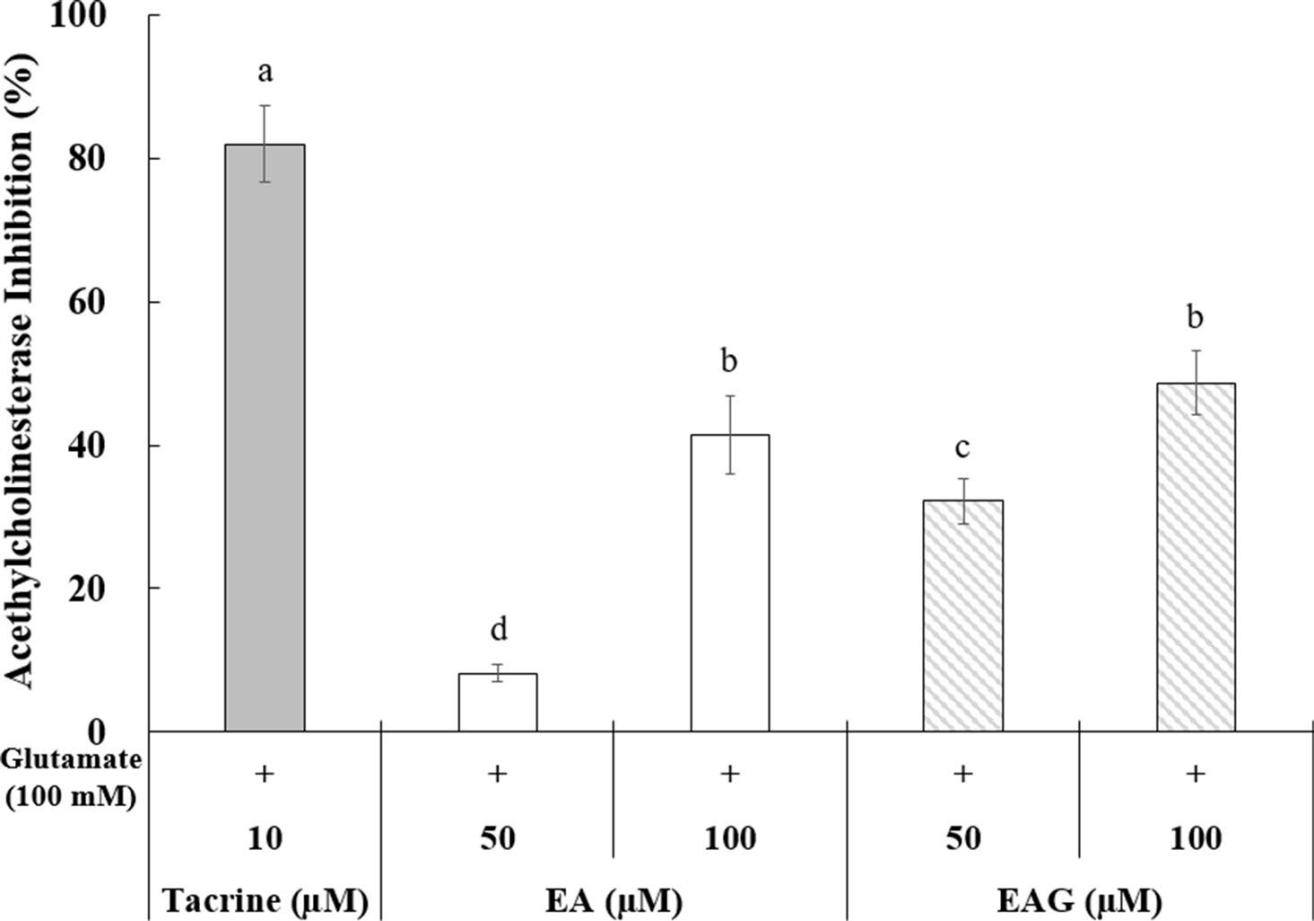
Functional evaluation of EA and EAG using SH-SY5Y cells. The morphological photograph showing that EA and EAG are pretreated to prevent cell damage from glutamate treatment (**a**). Cell viability according to treatment with 1–100 μM of EA or EAG compared with only buffer treatment. As positive controls, 1 and 10 μM of theanine were treated (**b**). Cortisol content in the cells treated with 1 or 10 μM theanine, EA, or EAG before treatment of glutamate (**c**). Inhibition rate of AChE enzyme after treatment with 100 mM glutamate followed by treatment with 10 μM tacrine, 50 or 100 μM of EA or EAG (**d**). Mean with same letter in each column are not significantly different by Duncan’s multiple range test (*p* < 0.05)

The inhibition of SH-SY5Y brain nerve cells growth by ellagic acid and its glucoside at the concentration range of 1.5–200 µM was evaluated. They did not inhibit cell growth with over 85% cell viability of the cells (Additional file 1: Fig. S1). The experiment of brain cell protective effect of ellagic acid glucoside using SH-SY5Y brain nerve cells showed 2–58% higher cell viability than the effect of ellagic acid at the same concentration. Compared with 10 μM theanine well-known to have brain-protective effect, 50 μM ellagic acid glucoside showed about 80% of cell viability. In addition, the cell viability of 100 μM ellagic acid was not statistically significant with that of 10 μM theanine (Fig. 5a, b).

Cortisol content in cells treated with ellagic acid glucoside at 10 µM was 7.7 ng/mL, which was lower than that (20.2 ng/mL) in cells treated with ellagic acid at 10 μM. This result indicates that ellagic acid glucoside has greater anti-stress effect than ellagic acid (Fig. 5c).

Furthermore, the inhibition rate for AChE enzymes known to degrade acetylcholine, a neurotransmitter, was measured to find out the anti-dementia effect. Compared to 10 μM tacrine commonly used to treat degenerative brain diseases such as dementia by inhibiting AChE enzyme, ellagic acid glucoside showed 32% of AChE inhibition rate at a concentration of 50 μM. This rate was four times higher than that with ellagic acid at the same concentration (Fig. 5d).

## Discussion

Ellagic acid glucoside was synthesized by an acceptor reaction of glucansucrase with ellagic acid and sucrose via α-glycosidic linkage. A previous study has shown that the attachment of xylose and rhamnose to ellagic acid formed by chemical synthesis can increase the inhibition activity of biofilm formed by bacteria (Fontaine et al. 2017). Meanwhile, our studies derived an attachment of glucose to ellagic acid using only edible enzymes and sucrose. Therefore, it can be used in the processed food or cosmetics industry. Many previous studies have used glucansucrase obtained from *L. mesenteroides* to transfer glucose or sugar moiety to functional compounds. For example, caffeic acid-3-*O*-α-glucopyranoside was synthesized by binding glucose to caffeic acid using the enzyme (Nam et al. 2017a). Another study also showed that 44% of luteolin was converted to luteolin glucosides, of which 17% were luteolin-4′-*O*-α-d-glucopyranoside and 27% were luteolin-3′-*O*-α-d-glucopyranoside (Bertrand et al. 2006). Whether the glucose moiety could bind to the hydroxyl group of carbon 3′ or 4′ of ellagic acid can be predicted by looking at the structure of okicamelliaside. Okicamelliaside is a natural substance in which glucose is bound to carbon 4′ of 3,4-dioxoloellagic acid. It is found in camellia leaves (Onodera et al. 2010).

Previous studies have indicated that the binding of glucose or sugar moiety to a substance can decrease its antioxidant effect. For example, the attachment of fructose to hydroquinone can decrease its antioxidants activity (Seo et al. 2009). Also, gallic acid glucoside shows sevenfold lower antioxidants activity than gallic acid (Nam et al. 2017a, b).

Treatment with glutamate can cause damage to SHSY5Y cells, including a decrease of cell viability, an increase of lactate dehydrogenase (LDH) release, and alterations of morphological structures (Sun et al. 2010). Theanine, a well-known brain-protectant compound, was used as a positive control in the present study. According to a previous study, pretreatment of cells with l-theanine can significantly block rotenone- or dieldrin-induced nuclear damage (Cho et al. 2008). Another study has revealed that ellagic acid (0.01–10 μM) can significantly increase cell proliferation and GSH level while decreasing levels of reactive oxygen species (ROS), MDA, TNF-α,β-galctosidase, and advanced glycation end products following d-galactose induced aging (Rahimi et al. 2018).

Cortisol as a steroid is widely known as the body’s stress hormone. If human body is exposed to an internal or external stressor, cortisol is released from the adrenal cortex. It allows the body to continue to stay on high alert (Thau and Sharma 2020). In a previous study, saliva cortisol level in overweight people is inhibited by 22.7% after 12-week of ellagic acid treatment (Liu et al. 2018). In the present study, cells treated with ellagic acid glucoside showed higher cortisol inhibition than those treated with ellagic acid at the same concentration.

Inhibition of cholinesterases is important in order to fight against Alzheimer’s disease. A previous study has shown that ellagic acid has a greater AChE inhibitory activity than other natural products such as quercetin, rutin, and chlorogenic acid (Neagu et al. 2015). The present study showed that ellagic acid glucoside had higher AChE inhibition effect than its precursor compound. Thus, ellagic acid glucoside could be used as a functional component for brain protection.

## Supplementary Information

**Additional file 1: Figure S1.** MTT assay was performed to evaluate the potential cytotoxic activity. SH-SY5Y cells were treated with different concentrations of ellagic acid or ellagic acid glucoside (1.5–200 μM). Results are presented as mean ± standard deviation of triplicate repeats. ∗, ∗∗: Significantly different from the control group at *p* < 0.05 and *p* < 0.01, respectively. **Table S1.** Independent variables, levels, and experimental codes used in response surface methodology (RSM). **Table S2.** ANOVA for RSM parameters fitted to second-order polynomial equations.

## Data Availability

Not applicable.

## Abbreviations

EA: Ellagic acid
EAG: Ellagic acid glucoside
LC–MS/MS: Liquid chromatography tandem mass spectrometry
AChE: Acetylcholinesterase
MTT: 3-(4,5-Dimethylthiazol-2-yl)-2,5-diphenyl-tetrazolium bromide
DPPH: 1,1-Diphenyl-2-picrylhydrazyl
TLC: Thin-layer chromatography
HPLC: High-pressure liquid chromatography
PDA: Photodiode array detector
RSM: Response surface methodology
CCD: Central composite design
MAb: Monoclonal antibody
