# Supplementary material for: Synthesis of ellagic acid glucoside using glucansucrase from Leuconostoc and characterization of this glucoside as a functional neuroprotective agent
Source: AMB Express. 2021 Jul 21;11:108. doi: 10.1186/s13568-021-01265-x (PMC8295447; doi:10.1186/s13568-021-01265-x)
Supplement: Supplementary file 1 — Additional file 1: Figure S1. MTT assay was performed to evaluate the potential cytotoxic activity. SH-SY5Y cells were treated with different concentrations of ellagic acid or ellagic acid glucoside (1.5–200 μM). Results are presented as mean ± standard deviation of triplicate repeats. ∗, ∗∗: Significantly different from the control group at p < 0.05 and p < 0.01, respectively. Table S1. Independent variables, levels, and experimental codes used in response surface methodology (RSM). Table S2. ANOVA for RSM parameters fitted to second-order polynomial equations. [file 13568_2021_1265_MOESM1_ESM.docx]

**Synthesis of ellagic acid glucoside using glucansucrase from *Leuconostoc* and characterization of this glucoside as a functional neuroprotective agent**

Hyejin Yu^1†^, Hana Jeong^1†^, Kwang-Yeol Yang^2^, Jeong-Yong Cho^3^, In Ki Hong^4^, Seung-Hee Nam ^1, 3*^

**Fig. S1** MTT assay was performed to evaluate the potential cytotoxic activity. SH-SY5Y cells were treated with different concentrations of ellagic acid or ellagic acid glucoside (1.5~200 μM). Results are presented as mean ± standard deviation of triplicate repeats.

∗, ∗∗: Significantly different from the control group at *p* < 0.05 and *p* < 0.01, respectively.

**Table S1** Independent variables, levels, and experimental codes used in response surface methodology (RSM)^a^.

**Table S2** ANOVA for RSM parameters fitted to second-order polynomial equations.


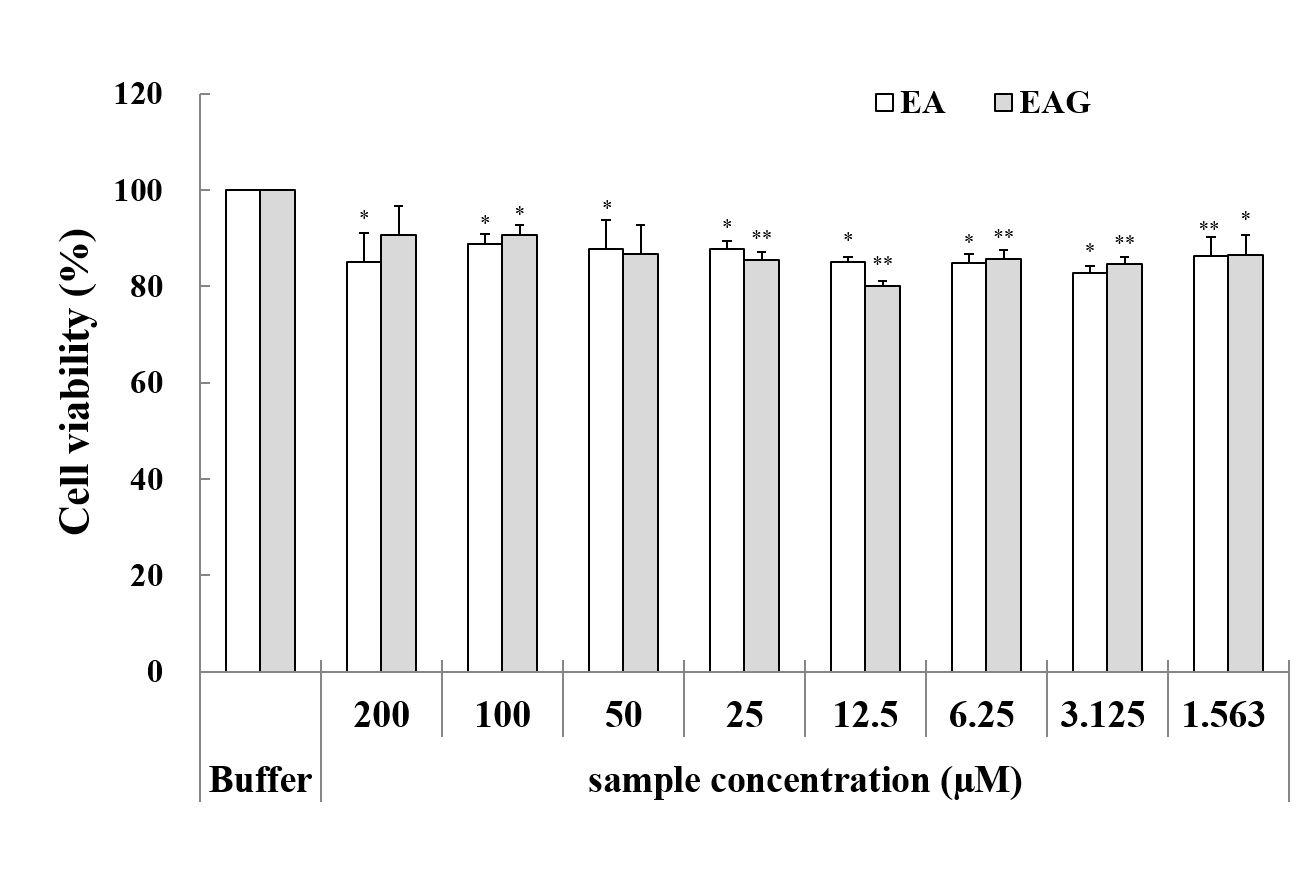


**Fig. S1**

**Table S1.** Independent variables, levels, and experimental codes used in response surface

methodology (RSM)^a^.

| Variables | Units | Symbol code | Levels | | | | |
| --- | --- | --- | --- | --- | --- | --- | --- |
|  |  |  | ‐ 1.682^b^ | ‐ 1 | 0 | + 1 | + 1.682^b^ |
| Sucrose | mM | x_1_ | 10.2 | 150 | 355 | 560 | 699.8 |
| Enzyme | (mU/mL) | x_2_ | 61.4 | 300 | 650 | 1000 | 1238.6 |
| Ellagic acid | mM | x_3_ | 1.5 | 5 | 10 | 20 | 25 |

^a^ *Y* = β_0_ + β_1_x_1_ + β_2_x_2_ + β_3_x_3_ + β_11_x_1_^2^ + β_22_x_2_^2^ + β_33_x_3_^2^ + β_12_x_1_x_2_ + β_13_x_1_x_3_ + β_23_x_2_x_3_.

^b^ Based on program design value

**Table S2.** ANOVA for RSM parameters fitted to second-order polynomial equations.

| Source | Sum of squares | Degree of freedom | Mean square | *F*‐value | *P*‐value > *F* |
| --- | --- | --- | --- | --- | --- |
| Model | 27.72 | 9 | 3.08 | 4.95 | < 0.0099 |
| Residual | 6.22 | 10 | 0.62 |  |  |
| Lack of fit | 5.5 | 5 | 1.1 | 7.63 | 0.0218 |
| Pure error | 0.72 | 5 | 0.14 |  |  |
| Cor Total | 33.94 | 19 |  |  |  |

Standard Deviation = 0.79, R^2^ = 0.82, C.V. = 38.55, Adj‐R^2^ = 0.65.
